# A Prospective, Phase I/II, Open-Label Pilot Trial to Assess the Safety of Hyperthermic Intraperitoneal Chemotherapy After Oncological Resection of Pancreatic Adenocarcinoma

**DOI:** 10.1245/s10434-021-10187-8

**Published:** 2021-06-15

**Authors:** Can Yurttas, Philipp Horvath, Imma Fischer, Christoph Meisner, Silvio Nadalin, Ingmar Königsrainer, Alfred Königsrainer, Stefan Beckert, Markus W. Löffler

**Affiliations:** 1 Department of General, Visceral and Transplant Surgery, University Hospital Tübingen, Tübingen, Germany; 2 Institute for Clinical Epidemiology and Applied Biometry, University Hospital Tübingen, Tübingen, Germany; 3 Department of General, Visceral and Thoracic Surgery, Landeskrankenhaus Feldkirch, Feldkirch, Austria; 4 German Cancer Consortium (DKTK) and German Cancer Research Center (DKFZ), Partner Site Tübingen, Tübingen, Germany; 5 Cluster of Excellence iFIT (EXC2180) ‘Image-Guided and Functionally Instructed Tumor Therapies’, University of Tübingen, Tübingen, Germany; 6 Department of General and Visceral Surgery, Schwarzwald-Baar Klinikum Villingen-Schwenningen, Villingen-Schwenningen, Germany; 7 Interfaculty Institute for Cell Biology, Department of Immunology, University of Tübingen, Tübingen, Germany; 8 Department of Clinical Pharmacology, University Hospital Tübingen, Tübingen, Germany

## Abstract

**Background:**

Pancreatic ductal adenocarcinoma (PDAC) is a common fatal disease with unfavorable prognosis, even after oncological resection. To improve survival, adding hyperthermic intraperitoneal chemotherapy (HIPEC) has been suggested. Whether HIPEC entails disproportional short-term mortality is unknown and a prospectively determined adverse events profile is lacking. Since both pancreatic resection and HIPEC may relevantly influence morbidity and mortality, this uncontrolled single-arm, open-label, phase I/II pilot trial was designed to assess the 30-day mortality rate, treatment feasibility, and adverse events connected with HIPEC after oncological pancreatic surgery.

**Methods:**

This trial recruited patients scheduled for PDAC resection. A sample size of 16 patients receiving study interventions was estimated to establish a predefined margin of treatment-associated short-term mortality with a power of > 80%. Patients achieving complete macroscopic resection received HIPEC with gemcitabine administered at 1000 mg/m^2^ body surface area heated to 42 °C for 1 hour.

**Results:**

Within 30 days after intervention, no patient died or experienced any adverse events higher than grade 3 that were related to HIPEC. Furthermore, treatment-related adverse events were prospectively documented and categorized as expected or unexpected. This trial supports that the actual mortality rate after PDAC resection and HIPEC is below 10%. HIPEC treatment proved feasible in 89% of patients allocated to intervention. Pancreatic fistulas, as key complications after pancreas surgery, occurred in 3/13 patients under risk.

**Conclusion:**

Combined pancreas resection and gemcitabine HIPEC proved feasible and safe, with acceptable morbidity and mortality. Based on these results, further clinical evaluation can be justified.

**Registration Number:**

NCT02863471 (http://www.clinicaltrials.gov).

**Supplementary Information:**

The online version contains supplementary material available at 10.1245/s10434-021-10187-8.

Pancreatic ductal adenocarcinoma (PDAC) is a common fatal disease, predominantly diagnosed in advanced stage^1^ and therefore leaves only very limited curative treatment options. The only curative treatment for PDAC to date remains radical surgical resection, but most patients do not qualify for this treatment.^2^,^3^ Unfortunately, even after surgery, long-term outcomes remain poor and, for example, radical macroscopic resection produced 5-year survival rates of approximately 10% in a randomized controlled trial,^4^ which leaves a substantial unmet medical need for effective adjunct treatments in this indication. Among the suggested root causes for surgical failure are local recurrence and peritoneal dissemination.^5^ To eliminate remaining cancer cells, hyperthermic intraperitoneal chemotherapy (HIPEC) with gemcitabine has been suggested, although this treatment has never been prospectively evaluated.^6^,^7^

This treatment approach is supported by preclinical studies where normothermic intra-abdominal gemcitabine administration was shown to prevent peritoneal metastasis in mice.^8^ According to first-in-human pharmacokinetic studies, intraperitoneal administration of gemcitabine is well-tolerated, with provisional data suggesting low toxicity and a manageable adverse event profile, even when left in situ for 24 hours.^9^,^10^ Preliminary evidence from 21 patients after PDAC resection suggested that gemcitabine HIPEC might prove effective to prevent peritoneal metastasis.^11^ In this study however, associated morbidity and mortality rates (at 9.5%) were substantial. In oncological pancreas surgery, acute mortality is also common^12^ and high 30-day mortality rates have been reported,^13^ as well as considerable morbidity rates (of up to 60%).^14^

HIPEC treatment, which is usually combined with cytoreductive surgery (CRS), may also involve increased morbidity and mortality, e.g. in peritoneal metastasis from colorectal cancer where morbidity rates between 23 and 45% were reported, along with acute mortality ranging from 0 to 12%.^15^

In spite of the high unmet medical need and preliminary preclinical and clinical evidence encouraging pre-emptive HIPEC for PDAC,^7^ as well as premature reports of efficacy,^11^ robust data supporting any wider clinical application remain absent. The PanHIPEC trial was therefore designed as a non-randomized, single-arm pilot trial aiming to prospectively investigate 30-day mortality and to establish an adverse events profile, as well as the feasibility of HIPEC in patients after complete oncological PDAC resection to manage the risk of implementing this treatment for possible further clinical development.

## Methods

### Trial Design

The PanHIPEC trial was prospectively designed as an uncontrolled, open label, single-arm pilot study, enrolling patients with imaging signs of non-metastatic PDAC scheduled for elective surgery. The primary study endpoint was to assess the 30-day mortality of participants after oncological surgery and HIPEC; a 30-day mortality rate of 10% was defined as a benchmark based on contemporary literature^12^,^13^,^15^ and considerations of risk–benefit. Secondary endpoints included assessment of safety and feasibility of treatment. Patients were interviewed and examined for severe adverse events, categorized according to the National Cancer Institute Common Terminology Criteria for Adverse Events (NCI CTCAE) version 4.0. As a trial-stopping rule, the occurrence of more than one death within 30 days after HIPEC was set. Since this trial was not designed to assess any oncological outcomes, respective results and survival data should be considered as exploratory and are only provided as a supplement.

### Trial Approval and Consent to Participate

The clinical trial was conducted in accordance with the principles of the Declaration of Helsinki as well as all applicable laws and regulations, and approval was obtained from the local Institutional Review Board (Project No. 426/2015AMG1) and the Federal German Institute for Drugs and Medical Devices (BfArM). The trial was registered with ClinicalTrials.gov (identifier: NCT02863471) and the European Union Clinical Trials Register (EudraCT Number 2015-002288-41) and conforms to International Committee of Medical Journal Editors (ICMJE) and Consolidated Standards of Reporting Trials (CONSORT) guidelines, where applicable. All patients (*n* = 20) gave appropriate informed consent, documented in writing, with full understanding of the experimental procedures prior to study enrollment and interventions.

### Inclusion and Exclusion Criteria

Patients aged ≥ 18 years with a Karnofsky performance status ≥ 70% (≙ Eastern Cooperative Oncology Group [ECOG] status ≤ 1^16^), with suspected PDAC according to imaging findings, but without evidence for distant metastasis and assessed as oncologically resectable, were allocated to treatment.

Exclusion criteria included participation in competing interventional trials, secondary malignant disease within 5 years before enrollment (except for basal cell carcinoma and curatively treated cervix carcinoma in situ), prior cytostatic therapy, or contraindications/hypersensitivity to gemcitabine. Patients deemed inoperable due to severe secondary illness, e.g. severe heart failure, coronary artery disease, arrhythmias or hypertension, and pulmonary or renal impairments, were excluded. Contraception standards for interventional clinical trials applied to this study, excluding pregnant or lactating women as well as men or women of childbearing potential not consenting to pre-emptive use of contraception measures. Before informed consent and study inclusion, a full medical history was taken, complete physical examination and laboratory testing were performed, and radiological scans evaluated. For women of childbearing potential, negative pregnancy tests were mandatory.

### Surgical Treatment and Hyperthermic Intraperitoneal Chemotherapy

After laparotomy, the abdomen was inspected for metastasis to the liver, non-locoregional lymph nodes, or the peritoneum, as well as other factors precluding full oncological resection. A tumor biopsy and intrasurgical frozen section analysis were required to confirm the diagnosis. Subsequent resection and reconstruction was performed according to disease location and extent, and closed HIPEC^17^ with gemcitabine 1000 mg/m^2^ body surface area^18^ was administered. Gemcitabine is an approved drug but is not labeled for the described use. The sutured abdomen was filled with up to 5.0 L of 0.9% saline, heated to 42 °C, and drugs added and circulated for 60 min (Performer HT, RanD Biotech, Medolla, Italy). Afterwards, the abdomen was flushed with 6.0 L of saline and reopened for definite wound closure after exploration.

### Evaluated Patient Cohort and Follow-Up

Enrolled patients allocated to HIPEC comprise the intention-to-treat (ITT) group, whereas the modified ITT (mITT) group encompasses only patients with an intraoperatively confirmed PDAC diagnosis, where an oncological resection was achieved and study treatment was applied. Only mITT patients were followed for survival and secondary endpoints. Prospective patient assessment included patient history and daily physical examinations and interviews, enquiring for predefined expected adverse events connected with gemcitabine. During hospitalization, daily laboratory tests encompassed automated differential blood counts, electrolytes, retention values, hepatic transaminases, cholestasis parameters, α-amylase, and lipase; α-amylase and lipase were also determined on a daily basis from abdominally inserted drains. At discharge before day 30 postsurgery, follow-up was continued by telephone; after day 30 postsurgery, a concluding end-of-trial visit with interviews and laboratory and physical examinations was performed.

### Statistics and Sample Size Determination

Thirty-day mortality was prospectively defined as the primary endpoint for statistical evaluation. A one-sided exact 95% confidence interval (CI) and a one-sided exact binomial test were used to evaluate the null hypothesis that the observed 30-day mortality rate is ≥ 10%, against the alternative that the 30-day mortality rate is < 10%. With a required number of 16 patients, the probability (power in terms of the exact binomial probability) of experiencing a critical incident (death within 30 days after surgery) for at least one patient is 81.5%, if the incidence of critical events is ≥ 10% in the main population. For statistical analysis, the exact one-sided binomial test was used.

### Assessment of Endpoints

As the primary endpoint, the incidence of death within the mITT population (with 95% CI) was analyzed. Additionally, the probability of 30-day survival was compared with the minimal acceptable probability of *π*_0_ = 0.9 using the one-sided binomial test.

Secondary endpoints included adverse events and feasibility of treatment. Feasibility was assessed as the frequency of patients receiving HIPEC among the ITT group. Patients were interviewed and examined for adverse events occurring within 30 days after HIPEC, which were categorized as expected adverse events or unexpected adverse events and graded according to CTCAE version 4.0. Gemcitabine-related expected adverse events were predefined and deduced from intravenous gemcitabine administration^19^ as neutropenia, thrombocytopenia, nausea, vomitus, diarrhea, stomatitis, and hair loss. Surgical complications ascribable to pancreatic surgery were specified in the study protocol and omitted among adverse events. Pancreatic fistulas as key surgical complications were documented and graded according to the classification of postoperative pancreatic fistulas (POPF) of the International Study Group of Pancreatic Surgery (ISGPS).^20^

### Exploratory Analyses

Overall survival (OS) of patients was assessed using Kaplan–Meier and Cox regression analysis, as well as log-rank analyses, and plotted using SPSS version 24 (IBM Corporation, Armonk, NY, USA). Further graphs were plotted using GraphPad Prism version 8 (GraphPad Software, La Jolla, CA, USA). Long-term follow-up for exploratory survival analyses was conducted by telephone. Due to the exploratory nature of these analyses, the respective information is provided as a supplement.

## Results

### Patient Characteristics, Screening, and Study Enrollment

**Fig. 1.**
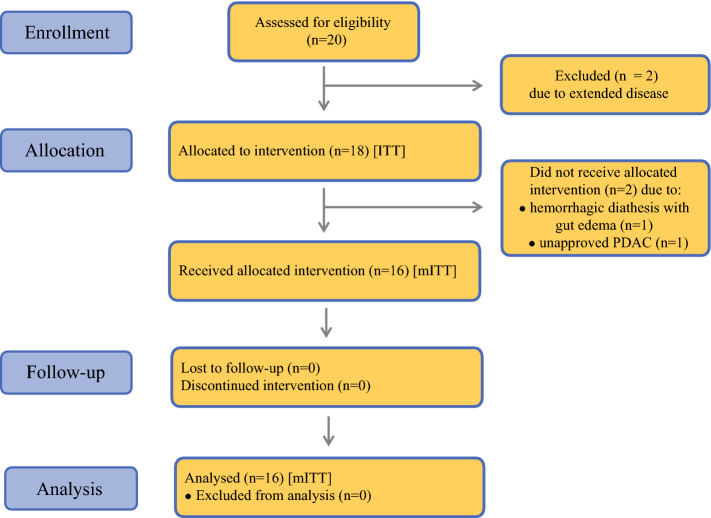
Enrollment, allocation, follow-up, and analysis of patients during the trial (the layout was adapted from the CONSORT 2010 statement).^43^*ITT* intention-to-treat group, *mITT* modified intention-to-treat group, *PDAC* pancreatic ductal adenocarcinoma

Between December 2015 and July 2017, 20 patients [13 men and 7 women, median age 62.5 years (range 50–79 years); median body surface area 1.8 m^2^ (range 1.5–2.3)] were assessed for eligibility. Of 20 screened patients scheduled for surgery, two patients were excluded due to extended disease. Among the remaining patients (*n* = 18; ITT), two patients were excluded from HIPEC during surgery—one patient due to histologically unconfirmed PDAC diagnosis and the other for severe hemorrhagic diathesis with edema of the gut. Ultimately, a total of 16 patients with confirmed PDAC diagnosis received HIPEC after oncological resection (mITT; *n* = 16) (Fig. 1). Patient characteristics, tumor staging and treatment details are provided in Table 1.

**Table 1.** Patient characteristics, tumor staging, and treatment

	Surgery with HIPEC (mITT) [*n* = 16]
Ethnicity	
Caucasian	16
Sex	
Female	5
Male	11
Median age, years (IQR)	62.5 (55.0–67.5)
Karnofsky Index	
100%	13
90%	2
80%	1
Location of tumor	
Head	10
Body	2
Tail	2
Unassignable	2
Tumor extension	
T1	0
T2	7
T3	9
T4	0
Nodal status	
N0	3
N1	10
N2	3
Metastasis	
M0	16
M1	0
Stage	
IA	0
IB	3
IIA	0
IIB	9
III	4
IV	0
Grade of differentiation	
G1	0
G2	7
G3	7
G4	2
Type of resection	
PPD	10
DP	3
TP	3
Resection status	
R0	6
R1	10
Median duration of surgery, min (IQR)	426 (339–498)
Median duration of hospital stay, days (IQR)	16 (13–18)

Data are expressed as *n* unless otherwise specified and are tabulated for the mITT group

Resection status was determined during postoperative histopathological work-up

TNM classification and staging is provided according to the UICC classification of malignant tumors, 8th edition

*DP* distal pancreatectomy, *HIPEC* hyperthermic intraperitoneal chemotherapy, *IQR* interquartile range, *mITT* modified intention-to-treat group, *PPD* partial pancreatoduodenectomy, *TP* total pancreatectomy, *UICC* Union for International Cancer Control

### Description of the Intention-to-Treat (ITT) and Modified ITT (mITT) Populations

Surgical procedures in the ITT group (*n* = 18) included partial pancreatoduodenectomy in 11 patients, distal pancreatectomy and splenectomy in 4 patients, and total pancreatectomy and splenectomy in 3 patients. Overall, 16/18 patients (mITT group) received HIPEC (details are provided in Table 2). In all patients in the mITT group, the PDAC diagnosis initially made from intrasurgical frozen sections was affirmed by the subsequent definite histological examination. Microscopic disease (R1) remained in 10 of these patients, whereas the resection margins were assessed as tumor-free (R0) in only 6/16 patients.

**Table 2.** HIPEC characteristics

Parameters	
Total administered drug dose, mg [median (IQR)]	1890 (1800–1930)
Volume of carrier solution, L	5.0
Drug concentration, mg/L	378 (360–386)
Temperature, °C [median (IQR)]	41.5 (41.5–41.9)
Flow rate, mL/min [median (IQR)]	1250 (1200–1285)
Duration, min	60

HIPEC treatment was performed with gemcitabine administered at 1000 mg/m^2^ BSA in the modified intention-to-treat group (*n* = 16)

*HIPEC* hyperthermic intraperitoneal chemotherapy, *BSA* body surface area, *IQR* interquartile range

### Mortality, Safety, and Feasibility

**Fig. 2.**
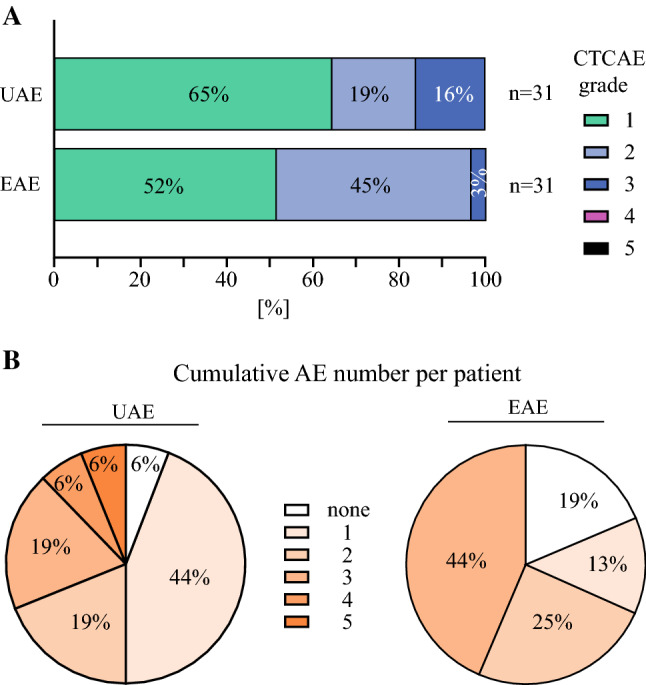
Adverse event (AE) overview in the mITT group. **a** Proportional distribution (%) of AEs classified according to National Cancer Institute CTCAE v.4.0, categorized as EAEs (*bottom column*) or UAEs (*top column*) [please see the Methods section (Assessment of Endpoints) for respective details]. **b** Proportional distribution (%) of patients experiencing (*n*) cumulative UAEs (*left*)/EAEs (*right*) over 30 days post-interventional follow-up. *mITT* modified intention-to-treat group, *UAEs* unexpected adverse events, *EAEs* expected adverse events, *CTCAE* Common Terminology Criteria for Adverse Events, *AE* adverse event

In the mITT group, no postoperative mortality was observed within the first 30 days (95% CI 0–0.172; *p* = 0.091), and the primary endpoint of the trial was therefore reached. None of the patients experienced any documented adverse events higher than grade 3 (Fig. 2a). Overall, 62 adverse events were documented, of which half (*n* = 31) were categorized as expected adverse events and the other half were categorized as unexpected adverse events (see Table 3). Most adverse events were categorized as mild (grade 1; 36/62), about one-third were categorized as moderate (grade 2; 20/62), and others were categorized as severe (grade 3; 6/62). The maximum documented severity of expected adverse events was grade 3 (severe), affecting one patient. In addition to these adverse events predefined as ‘expected’, a total of 31 unexpected adverse events affected 15 patients. The maximum extent of unexpected adverse events was also severe (grade 3), affecting four patients in whom five severe adverse events overall were documented. A relation of unexpected adverse events to the study intervention with HIPEC was assessed as probable or possible in two patients, whereas this was evaluated as unlikely in 12 occasions of unexpected adverse events. About half of the unexpected adverse events were considered not related to HIPEC treatment. Single patients were affected by up to three expected adverse events and five unexpected adverse events in the course of postoperative surveillance (Fig. 2b). When describing surgical complications according to the Clavien–Dindo Classification^21^, the maximum grade was IVb, affecting one patient with leakage of the pancreatic anastomosis with a septic shock requiring prolonged intensive care. Grade IIIa interventions for surgical site infection and myocardial infarction were necessary in three patients. Overall, the majority of adverse events were considered as the minimal deviation from postoperative course (i.e. grade I) or requiring pharmacologic treatment (grade II) (see Table 3). Feasibility was assessed as the percentage of patients with histologically confirmed PDAC receiving HIPEC after oncological resection (*n* = 16) among all patients allocated to intervention (*n* = 18). This fraction included 89% of patients.

**Table 3.** Treatment-emergent expected adverse events (EAEs) and unexpected adverse events (UAEs)

EAEs	No. of affected patients	Maximum grade CTCAE / (Clavien–Dindo)
Nausea	12	3 (I)
Vomiting	9	2 (I)
Diarrhea	8	2 (I)
Stomatitis	1	1 (I)
Loss of hair	1	1 (I)

UAEs	No. of affected patients	Maximum grade CTCAE / (Clavien–Dindo)
Anemia	4	3 (I)
Atrial fibrillation	2	3 (II)
Tachycardia	1	3 (I)
Surgical site infection	2	3 (IIIa)
Pneumonia	2	3 (II)
Pulmonary embolism	1	2 (II)
Myocardial infarction	1	2 (IIIa)
Pleural effusion	5	1 (II)
Exanthema	2	1 (I)
Fatigue	1	1 (I)
Dysgeusia	1	1 (I)
Inappetence	1	1 (I)
Exsiccosis	1	1 (I)
Obstipation	1	1 (I)
Back pain	1	1 (I)
Flank pain	1	1 (I)
Fever	1	1 (I)
Leukopenia	1	1 (I)
Urinary tract infection	1	1 (II)
Pancreatitis	1	1 (I)

AEs documented in the modified intention-to-treat group (*n* = 16) are classified as EAEs (*n* = 31) or UAEs (*n* = 31) and are organized according to maximum severity (CTCAE v.4.0) and number of patients affected, including corresponding grades according to the Clavien–Dindo classification (not foreseen in the trial protocol)

*EAEs* expected adverse events, *UAEs* unexpected adverse events, *CTCAE* Common Terminology Criteria for Adverse Events, *AEs* adverse events

### Pancreatic Fistulas

Pancreatic fistulas occurred in 3/13 patients under risk, omitting 3 patients in the mITT group who received a total pancreatectomy. Two patients were treated conservatively (both grade A according to the POPF classification), whereas one patient underwent a resection of the pancreatic anastomosis due to anastomotic leakage with consecutive septic shock and temporary organ failure, receiving external drainage of the pancreatic duct (grade C). Hereupon the patient slowly recovered and was discharged home 24 days after surgery. The patient experienced rapid progressive disease 2 months later and passed away after 21 months due to malignant obstruction ileus. All patients were treated according to implemented standards without any alterations due to HIPEC therapy.

### Post-Interventional Follow-Up and Mortality

After trial termination, patients included in the mITT group were further followed to determine OS after PDAC surgery and HIPEC for exploratory analysis. The median OS of this cohort was 16.1 months after surgery and the 1-year survival rate was 62.5% (*n* = 10) (electronic supplementary Fig. 1). Overall, no short-term mortality was evidenced, neither after 30 days nor 90 days after CRS and HIPEC. The first patient died 4.4 months after HIPEC due to tumor progression of an anaplastic PDAC. At the end of follow-up (approximately 24 months), five patients remained alive or of unknown status (electronic supplementary Table 1).

## Discussion

In spite of a theoretical rationale for performing HIPEC after PDAC resection,^6^,^7^ robust safety data are missing and potential downsides remain unknown. HIPEC was not only proposed as an adjunct treatment^6^,^7^ but has also already been administered to PDAC patients, both after oncological resection^7^,^11^,^22^ and following CRS for peritoneal metastasis.^23^ Although HIPEC has been shown to be comparably safe among high-risk surgical oncology procedures,^24^ evidence is lacking that a combination of pancreatic surgery and HIPEC does not cause disproportional cumulative mortality and major morbidity, surpassing its potential benefits. In many clinical indications where HIPEC is discussed, pilot studies formally assessing mortality and prospective adverse event profiles are lacking regardless of their broad clinical application.^25^ Recent work comparable with our study, performed in peritoneal metastasis from gastric cancer, has shown substantial treatment-related mortality, with 50% mortality at the highest investigated dose level of docetaxel in combination with oxaliplatin.^26^ Furthermore, serious adverse events were reported in 17/25 patients.

It is therefore against this background that we aimed to assess treatment-associated short-term mortality in a pilot trial, for combining both procedures. Interestingly, even in peritoneal metastasis, where pancreatic resection was considered a contraindication for CRS and HIPEC due to a high complication risk, recent data suggest it may be defensible.^27^ Based on risk–benefit considerations and mortality rates from the literature for both procedures,^12^,^13^,^15^ a benchmark was set at 10% 30-day mortality, assuming that for the investigated clinical setting, mortality rates ≥ 10% could question further clinical development. Our study now suggests that HIPEC does not substantially contribute to acute mortality since the mortality of the combined procedure was determined below 10% due to the absence of deaths during the observation period (30 days after PDAC surgery and HIPEC; primary endpoint). Therefore, we conclude that adding gemcitabine HIPEC to pancreatic resection can be considered sufficiently safe in experienced medical centers.

Previously consolidated adverse event profiles for HIPEC with gemcitabine were unavailable and hitherto only anecdotal data were accessible, e.g. Sugarbaker et al. merely reported no grade 3 or 4 toxicities^7^ and Tentes et al. refer to one case of afebrile neutropenia (grade 2).^11^ We have witnessed expected adverse events and previously undescribed adverse events in all but one patient in the immediate postoperative course with a maximum of grade 3. Such severe adverse events were reported in six instances, rendering the adverse event profile acceptable in our view. Pancreatic fistulas are reported as frequent complications after pancreas surgery, particularly when combined CRS and HIPEC is performed,^28^ frequently involving severe complications and potentially increasing mortality.^27^ Such complications occurred in three patients in our study cohort (two grade A and one grade C according to the POPF classification^20^), among 13 patients under risk. These results are comparable with respective complication rates reported in the literature after pancreas surgery;^29^ however, since 3 of the 16 patients in the mITT cohort received a total pancreatectomy, this may have reduced the overall risk profile and special attention should be paid to the treatment-associated risk for POPF in any future trials. On follow-up after trial termination, there was no 90-day mortality and the first HIPEC patient died 4.4 months after surgery.

Due to the early stage of development and the small cohort size, this prospective clinical trial comes with heavy limitations, particularly regarding any ancillary clinical or oncological endpoints, since this trial was not designed to reliably assess such endpoints (including OS, comparisons with established treatments, etc.). Therefore, the presented data have to be considered as exploratory and without (immediate) relevance for patient care. Of note, whether HIPEC proves effective in PDAC by adding any benefits, such as increased long-term survival or disease control, remains to be answered. Further prevailing issues are, for instance, the remaining microscopic disease in most patients (*n* = 10) and that current adjuvant treatment options have not been used, as well as the highly variable mortality and morbidity observed among German centers^30^ that are influenced by patient- and treatment-intrinsic variables, a fact that ultimately precludes any valid assessment of oncological benefits in this case.

An immanent design aspect of this clinical trial is that no control group without HIPEC treatment has been included, which does limit the precise attribution of adverse events to specific parts of the investigated treatment. Furthermore, the heterogeneous patient cohort and surgical treatment may raise legitimate critique in the context of oncological benefits, whereas these circumstances do not interfere with the primary endpoints of this trial (see also van der Kaaij et al.^26^).

On the other hand, there are clear and defined benefits of this prospective clinical trial that include robust safety data, which hitherto were unavailable. It should also be appreciated that this is the first ever prospectively planned and conducted HIPEC trial in this indication. Considering unsystematic previous clinical tests of the approach (as described e.g. by Tentes et al.^22^), our robust trial design and predefined aims add validity and can therefore answer relevant clinical questions in a more definitive manner.

Because, for example, in colorectal cancer where HIPEC was evaluated after CRS^31^ as well as for the prevention of peritoneal metastasis^32^ the latest randomized controlled trials did not confirm a clinical benefit, an ongoing controversy has evolved regarding the role of the HIPEC treatment.^33^-^37^ A recent systematic review on intraperitoneal chemotherapy in PDAC patients could only identify data on 85 patients in eight publications. Based on these limited data, the treatment was concluded to be well tolerated and potentially suited for short-term disease control, whereas adjuvant HIPEC in resectable PDAC was discouraged.^38^ Although significant oncological effectiveness of HIPEC in PDAC may emerge as doubtful, such comprehensive conclusions based on very limited and preliminary evidence may likewise be questioned. Since HIPEC is influenced by a multitude of factors,^39^,^40^ preclinical research and modeling appears pivotal as a guide for the future.^37^,^41^,^42^ Nevertheless, we are convinced that this pilot trial has added relevant information, providing solid knowledge regarding adverse events to be expected with gemcitabine HIPEC, and fundamental evidence on mortality risks incurred when adding HIPEC after PDAC resection, essential facts that are required before any possible further clinical development should be considered.

## Supplementary Information

Below is the link to the electronic supplementary material.

SUPPLEMENTARY FIG. 1 Overall survival (OS) of patients after PDAC resection and HIPEC. Follow-up of PDAC patients successfully treated according to the study protocol with oncological resection and subsequent gemcitabine HIPEC (n = 16; i.e. modified intention-to-treat [mITT] group). Survival proportion was estimated using Kaplan–Meier regression analysis. (PNG 17 KB)

Supplementary file2 (PDF 88 KB)
