# Supplementary figures and images for: A Prospective, Phase I/II, Open-Label Pilot Trial to Assess the Safety of Hyperthermic Intraperitoneal Chemotherapy After Oncological Resection of Pancreatic Adenocarcinoma
Source: Ann Surg Oncol. 2021 Jun 15;28(13):9086–95. doi: 10.1245/s10434-021-10187-8 (PMC8205203; doi:10.1245/s10434-021-10187-8)

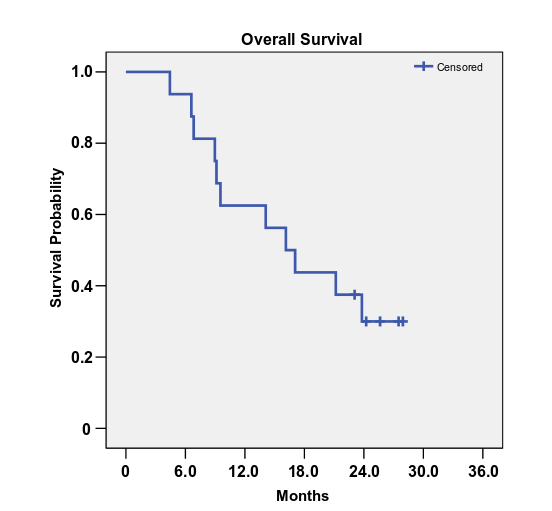

Supplement: Supplementary file 1 — SUPPLEMENTARY FIG. 1 Overall survival (OS) of patients after PDAC resection and HIPEC. Follow-up of PDAC patients successfully treated according to the study protocol with oncological resection and subsequent gemcitabine HIPEC (n = 16; i.e. modified intention-to-treat [mITT] group). Survival proportion was estimated using Kaplan–Meier regression analysis. (PNG 17 KB) [file 10434_2021_10187_MOESM1_ESM.png]
