# Supplementary material for: A Prospective, Phase I/II, Open-Label Pilot Trial to Assess the Safety of Hyperthermic Intraperitoneal Chemotherapy After Oncological Resection of Pancreatic Adenocarcinoma
Source: Ann Surg Oncol. 2021 Jun 15;28(13):9086–95. doi: 10.1245/s10434-021-10187-8 (PMC8205203; doi:10.1245/s10434-021-10187-8)
Supplement: Supplementary file 2 — Supplementary file2 (PDF 88 KB) [file 10434_2021_10187_MOESM2_ESM.pdf]

Suppl. Tab. 1: Extended patient follow-up data.

Patient follow-up was performed after trial termination. Due to this fact, respective data and all resultant analyses should be regarded as exploratory and with respective caution. Overall survival is annotated as the time span between the date of surgery and death or last recorded patient contact. Known time of death is annotated with Y (yes), otherwise N (no) is indicated. Progression-free survival (PFS) is recorded as the time to first documented cancer recurrence with respective site of recurrence if available or by last time of contact and n.a. in case the patients remained without recurrence.

OS: Overall survival; PFS: Progression-free survival; R0: microscopically complete resection; R1: residual disease in microscopy; Y - yes; N – no.; n.a. - not applicable; n.d. - no data available.

Pat. No.	OS (months)	Death Y/N	PFS (months)	Site of recurrence	Resection status
**1**	4.4	Y	n.d.	n.d.	R1
**2**	6.6	Y	n.d.	n.d.	R1
**3**	6.8	Y	n.d.	n.d.	R1
**4**	9.0	Y	n.d.	n.d.	R0
**5**	9.1	Y	6.0	Liver, Lung	R0
**6**	9.5	Y	1.3	Peritoneum	R1
**7**	14.1	Y	6.5	Peritoneum	R1
**8**	16.1	Y	3.0	Liver, Peritoneum	R0
**9**	17.1	Y	3.2	Liver	R1
**10**	21.2	Y	2.0	Local, Lymph node	R1
**11**	23.1	N	1.7	Local	R1
**12**	23.8	Y	6.1	Local, Liver, Peritoneum	R0
**13**	24.2	N	2.7	Local	R1
**14**	25.6	N	n.d.	n.a.	R0
**15**	27.5	N	27.5	n.a.	R0
**16**	27.9	N	7.4	Liver	R1
